# Utility of Hydro-Computed Tomography in Diagnosing Surgical Clip Migration into the Gastric Wall Post-splenectomy

**DOI:** 10.14309/crj.0000000000001908

**Published:** 2025-12-05

**Authors:** Ridhima Kaul, Arjun Chatterjee, Andrei Purysko, Tyler Stevens, Hassan Siddiki, Amit Bhatt, Kyungran Justina Cho

**Affiliations:** 1 Department of Internal Medicine, Cleveland Clinic Foundation, Cleveland, OH; 2 Department of Gastroenterology and Hepatology, Digestive Disease Institute, Cleveland Clinic, Cleveland, OH; 3 Imaging Institute, Cleveland Clinic Foundation, Cleveland, OH

**Keywords:** hydro-CT, gastric wall erosion, surgical clip migration, splenectomy, endoscopic removal

## CASE REPORT

**Figure 1.**
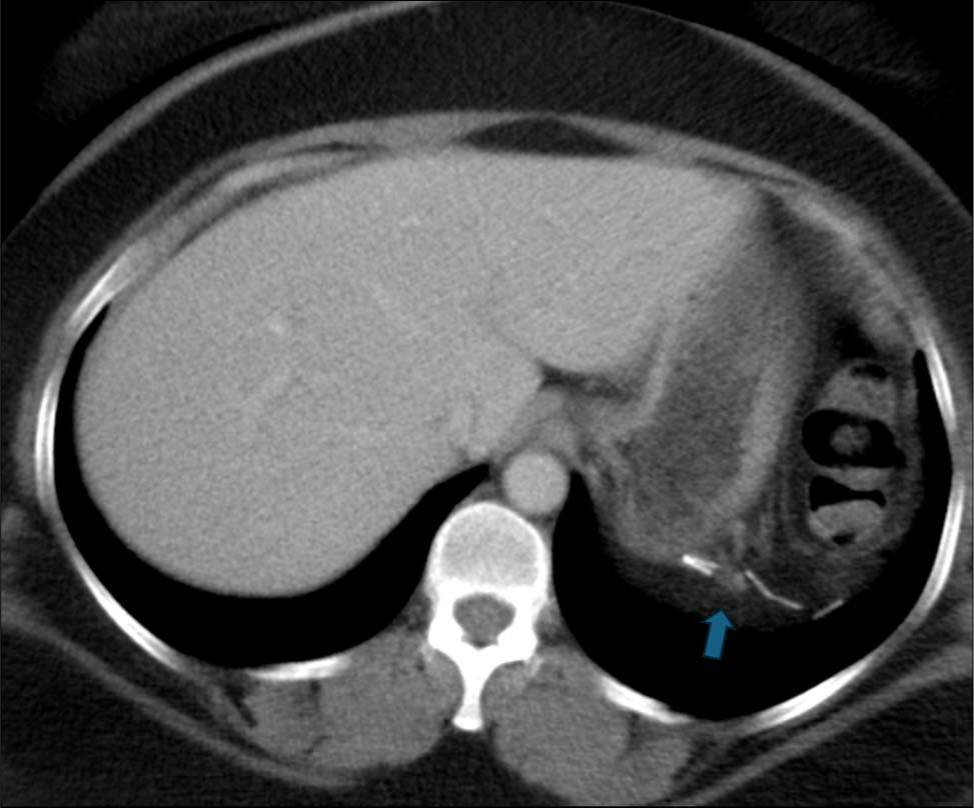
Axial computed tomography scan image of the abdomen and pelvis with intravenous contrast only shows splenectomy clips in the left upper quadrant abutting the stomach (blue arrow).

**Figure 2.**
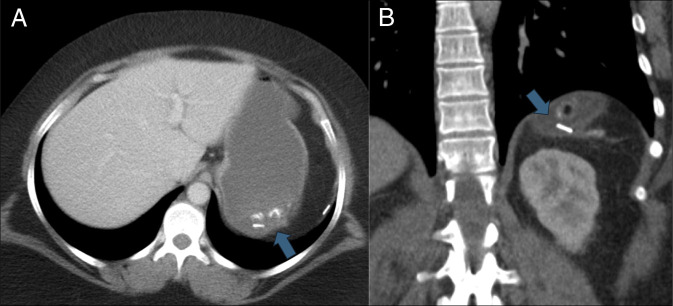
Axial (A) and coronal (B) computed tomography of the abdomen obtained with intravenous contrast and 900 mL of water by mouth show one of the splenectomy clips located inside the gastric lumen (blue arrow).

**Figure 3.**
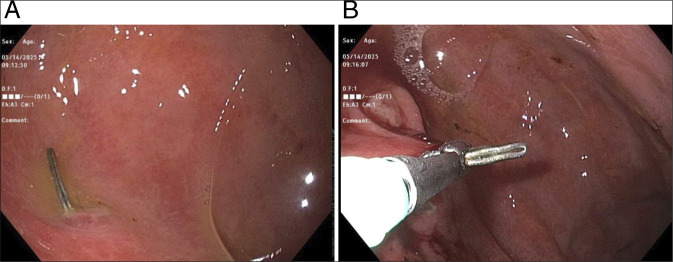
Upper endoscopy showing a surgical clip in the fundus of the stomach (A), which was successfully removed (B).

A 42-year-old woman presenting with a past medical history of systemic lupus erythematosus complicated by immune thrombocytopenic purpura, status post splenectomy, presented with 3 days of constant left sided chest pain. Cardiac workup was negative. Abdominal and pelvic computed tomography (CT) showed 2 surgical clips from her previous splenectomy located in the posterior wall of the stomach with potential erosion into the gastric wall, with associated focal thickening in the area of previous splenectomy (Figure 1). It was unclear from this imaging whether the surgical clip was extrinsic to the posterior gastric wall or had eroded into the posterior wall of the gastric fundus. Gastroenterology was consulted and an abdominal hydro-CT was recommended. This technique uses water to distend the stomach for better visualization of the gastric wall.^1–4^ The hydro-CT protocol showed a surgical clip within the lumen of the gastric fundus (Figure 2). She underwent upper endoscopy with removal of the surgical clip (Figure 3). Unlike a traditional abdominal and pelvic CT, the hydro-CT was able to precisely determine the location of the clip, highlighting the utility of the hydro-CT for better visualization of the gastric wall in the United States.^1–4^

## DISCLOSURES

Author contributions: R. Kaul and A. Chatterjee: Analysis of existing literature, interpretation, and drafting of the article; A. Purysko: Acquisition of radiologic images, editing, and reviewing the article; T. Stevens, H. Siddiki, A. Bhatt, KJ Cho: Acquisition of endoscopic images, editing, and reviewing the article and is the article guarantor.

Financial disclosure: None to report.

Informed consent was obtained for this case report.

## ABBREVIATION:

CT: Computed tomography
